# Prediction of pyrazinamide resistance in *Mycobacterium tuberculosis* using structure-based machine-learning approaches

**DOI:** 10.1093/jacamr/dlae037

**Published:** 2024-03-18

**Authors:** Joshua J Carter, Timothy M Walker, A Sarah Walker, Michael G Whitfield, Glenn P Morlock, Charlotte I Lynch, Dylan Adlard, Timothy E A Peto, James E Posey, Derrick W Crook, Philip W Fowler

**Affiliations:** Nuffield Department of Medicine, University of Oxford, John Radcliffe Hospital, Headley Way, Oxford OX3 9DU, UK; Nuffield Department of Medicine, University of Oxford, John Radcliffe Hospital, Headley Way, Oxford OX3 9DU, UK; Nuffield Department of Medicine, University of Oxford, John Radcliffe Hospital, Headley Way, Oxford OX3 9DU, UK; National Institute of Health Research Oxford Biomedical Research Centre, John Radcliffe Hospital, Headley Way, Oxford OX3 9DU, UK; NIHR Health Protection Research Unit in Healthcare Associated Infection and Antimicrobial Resistance, University of Oxford, Oxford, UK; Division of Molecular Biology and Human Genetics, Faculty of Medicine and Health Sciences, SAMRC Centre for Tuberculosis Research, DST/NRF Centre of Excellence for Biomedical Tuberculosis Research, Stellenbosch University, Tygerberg, South Africa; Division of Tuberculosis Elimination, National Center for HIV/AIDS, Viral Hepatitis, STD, and TB Prevention, Centers for Disease Control and Prevention, Atlanta, GA, USA; Nuffield Department of Medicine, University of Oxford, John Radcliffe Hospital, Headley Way, Oxford OX3 9DU, UK; Nuffield Department of Medicine, University of Oxford, John Radcliffe Hospital, Headley Way, Oxford OX3 9DU, UK; Nuffield Department of Medicine, University of Oxford, John Radcliffe Hospital, Headley Way, Oxford OX3 9DU, UK; National Institute of Health Research Oxford Biomedical Research Centre, John Radcliffe Hospital, Headley Way, Oxford OX3 9DU, UK; Division of Tuberculosis Elimination, National Center for HIV/AIDS, Viral Hepatitis, STD, and TB Prevention, Centers for Disease Control and Prevention, Atlanta, GA, USA; Nuffield Department of Medicine, University of Oxford, John Radcliffe Hospital, Headley Way, Oxford OX3 9DU, UK; National Institute of Health Research Oxford Biomedical Research Centre, John Radcliffe Hospital, Headley Way, Oxford OX3 9DU, UK; NIHR Health Protection Research Unit in Healthcare Associated Infection and Antimicrobial Resistance, University of Oxford, Oxford, UK; Nuffield Department of Medicine, University of Oxford, John Radcliffe Hospital, Headley Way, Oxford OX3 9DU, UK; National Institute of Health Research Oxford Biomedical Research Centre, John Radcliffe Hospital, Headley Way, Oxford OX3 9DU, UK

## Abstract

**Background:**

Pyrazinamide is one of four first-line antibiotics used to treat tuberculosis; however, antibiotic susceptibility testing for pyrazinamide is challenging. Resistance to pyrazinamide is primarily driven by genetic variation in *pncA*, encoding an enzyme that converts pyrazinamide into its active form.

**Methods:**

We curated a dataset of 664 non-redundant, missense amino acid mutations in PncA with associated high-confidence phenotypes from published studies and then trained three different machine-learning models to predict pyrazinamide resistance. All models had access to a range of protein structural-, chemical- and sequence-based features.

**Results:**

The best model, a gradient-boosted decision tree, achieved a sensitivity of 80.2% and a specificity of 76.9% on the hold-out test dataset. The clinical performance of the models was then estimated by predicting the binary pyrazinamide resistance phenotype of 4027 samples harbouring 367 unique missense mutations in *pncA* derived from 24 231 clinical isolates.

**Conclusions:**

This work demonstrates how machine learning can enhance the sensitivity/specificity of pyrazinamide resistance prediction in genetics-based clinical microbiology workflows, highlights novel mutations for future biochemical investigation, and is a proof of concept for using this approach in other drugs.

## Introduction

*Mycobacterium tuberculosis* is an evolutionarily ancient human pathogen that is the leading cause of death by infectious disease worldwide, except during the SARS-CoV-2 pandemic. In 2021, TB was responsible for 1.6 million deaths and 10.6 million new infections.^1^ TB control efforts have been hampered by the evolution of resistance to antibiotics, threatening the efficacy of the standard four-drug antibiotic regimen consisting of rifampicin, isoniazid, ethambutol and pyrazinamide. Pyrazinamide plays a critical role in TB treatment through its specific action on slow-growing, ‘persister’ bacteria, which often tolerate other drugs due to their reduced metabolism.^2–6^ Due to its unique sterilizing effect and its synergy with new TB drugs such as bedaquiline, pyrazinamide is also included in new treatment regimens targeting drug-resistant TB.^7–12^ Therefore, accurately and rapidly determining whether a clinical isolate is resistant to pyrazinamide is critically important for the treatment of TB.

Most culture-based laboratory methods to determine pyrazinamide resistance are technically challenging, requiring highly trained technicians. Even then, results are often not reproducible, meaning these methods are rarely employed in low-resource and/or high-burden clinical settings.^13^ Even the current gold standard, the Mycobacteria Growth Indicator Tube (MGIT), which is relatively simple to use, can suffer from low precision, with false-resistance rates of 1%–68% reported.^14–20^ As the prevalence of MDR and XDR TB increases, this lack of precision will become more of a problem.

Resistance to rifampicin or isoniazid can be predicted in most isolates (90%–95% and 50%–97%, respectively) by the presence of a small number of highly penetrant genetic variants in short and well-delineated regions of one or two genes (*rpoB* and *katG*/*fabG1*, respectively).^3^ However, despite pyrazinamide being used to treat TB since 1952, comparatively less is known about which genetic variants confer resistance compared with other first-line drugs.^4^ In the recent catalogue of resistance-associated mutations of *M. tuberculosis* published by the WHO, the performance for pyrazinamide was markedly lower (72.3% sensitivity and 98.8% specificity) than either rifampicin or isoniazid (93.8% and 98.2% or 91.2% and 98.4% sensitivity and specificity, respectively).^21,22^ While some of this poor performance is likely due to inaccuracies in phenotypic testing, a comprehensive genetic catalogue for pyrazinamide resistance mutations remains elusive.

**Figure 1.**
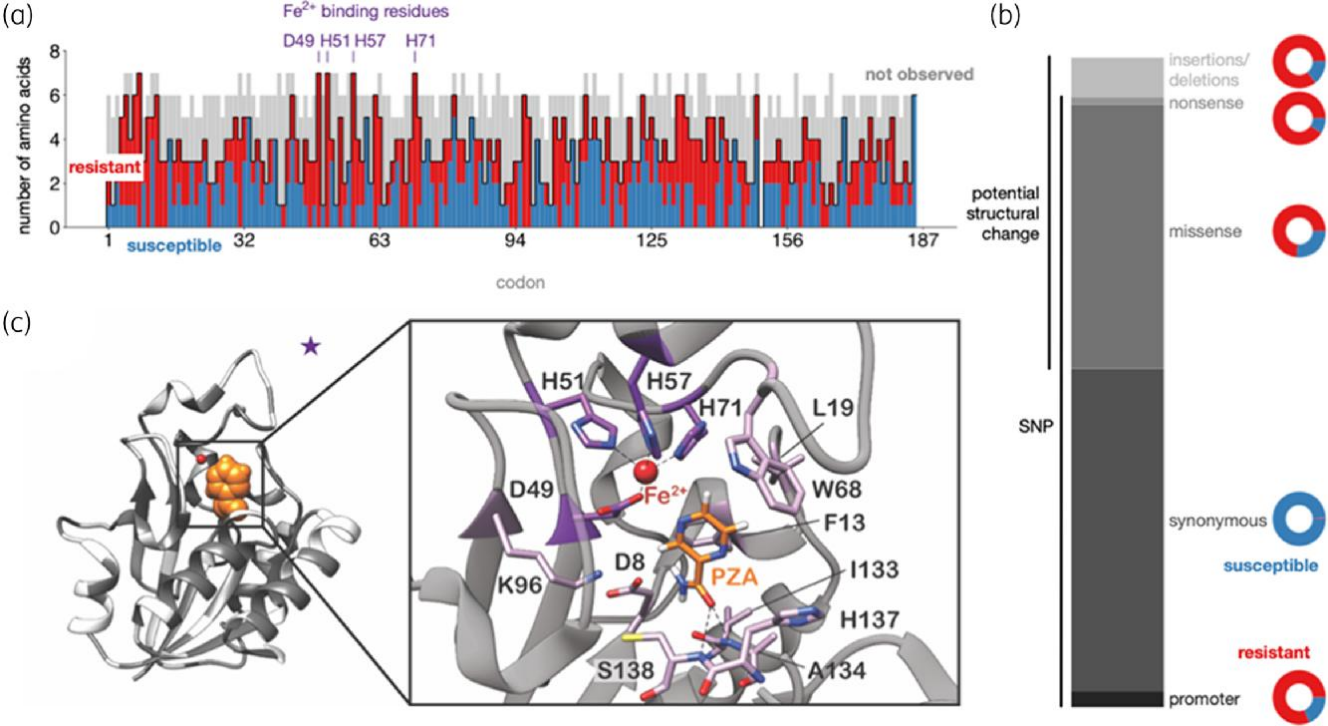
Distribution of PncA mutations from published datasets. (a) Barplot of the impact of possible missense mutations in PncA by amino acid position. High-confidence resistant (red) and susceptible (blue) mutations are overlaid on the possible missense mutations whose effect on resistance is unknown or unclear (grey). (b) Distribution of the types of mutations reported by the CRyPTIC consortium *et al*. (c) Missense mutations from the dataset plotted onto the PncA structure (PDB ID: 3PL1) in dark grey. A pyrazinamide molecule (orange) has been modelled into the active site.

Pyrazinamide is a pro-drug that is converted to its active form of pyrazinoic acid by the action of PncA, a pyrazinamidase/nicotinamidase encoded by the *pncA* gene.^23^ While other genetic loci have been implicated in pyrazinamide resistance (notably *rpsA*, *panD*, *clpC1*, and the putative efflux pumps *Rv0191*, *Rv3756c*, *Rv3008* and *Rv1667c*), the majority (70%–97%) of pyrazinamide-resistant clinical isolates harbour genetic variants in either the promoter region or coding sequence of *pncA.*^21,24–34^ In contrast to the well-delineated and relatively restricted ‘resistance-determining regions’ found in *rpoB* (rifampicin, 27 codons) and *katG* (isoniazid, single codon), pyrazinamide-resistant variants have been identified along the entire length of the *pncA* gene (Figure 1a) with no single variant predominating. Hence, while targeted genome sequencing or WGS approaches are capable of assaying the entire *pncA* gene, the number and diversity of resistance-conferring variants in *pncA* fundamentally limits the sensitivity and specificity of heuristic approaches that aim to predict the effectiveness of pyrazinamide based on a catalogue of previously observed genetic variants.^3,13,24,29,30,35,36^

Genetics-based clinical microbiology for TB currently depends on being able to *infer* the effect of any likely occurring *pncA* mutation on pyrazinamide susceptibility. Recent studies to identify pyrazinamide-resistance-determining mutations have focused on either classifying mutations from previously observed clinical isolates or discovering novel mutations through *in vitro/in vivo* screening approaches.^21,22,30,37–39^ However, these strategies are constrained, respectively, by the relative paucity of sequenced clinical isolates compared with the number of potential resistance-causing mutations and the lack of laboratory capacity to systematically generate and test mutants. Computational modelling approaches^40^ can potentially *predict* the effect of a significant number of missense mutations^41–44^ before they are observed in clinical isolates. Several studies have already trained machine-learning models on a number of anti-tuberculars,^45–48^ including pyrazinamide.^49^

As PncA is not essential and can be inactivated through defects in protein folding, reduced stability, distortion of active site geometry, abrogation of metal binding, or some combination of these, we expected a machine-learning approach to be ideally suited to simultaneously consider all these possible mechanisms of PncA inactivation, and hence more accurately predict pyrazinamide resistance/susceptibility. In this paper, we confirm using the largest *Train/Test* and *Validation* datasets used to date that machine-learning models that learn from a range of structural, chemical and evolutionary features can robustly and accurately predict the effect of missense amino acid mutations on pyrazinamide susceptibility.

## Materials and methods

We first constructed independent *Train*, *Test* and *Validation* datasets (Table 1). The first two were built by combining a comprehensive *in vitro*/*in vivo* mutagenesis study^38^ with two published catalogues of *M. tuberculosis* genetic variants associated with resistance,^21,39^ resulting in a *Train/Test* dataset of 664 non-redundant missense mutations (349 associated with resistance) where there was no discrepancy in the predicted phenotype. This was then split 70:30 to produce independent *Train* and *Test* datasets containing 464 and 200 mutations, respectively (Table 1). The *Validation* dataset was constructed by aggregating 24 231 clinical samples from three collections,^13,30,50^ resulting in 4027 samples containing one of 367 non-redundant missense PncA mutations. Briefly, phenotypes for strains with single missense mutations in *pncA* were aggregated by mutation, tallying the results of the phenotypic testing. Mutations that were resistant or susceptible at least 75% of the time and that had been phenotyped at least four times were included. Additionally, mutations that had been phenotyped at least twice with no discrepancies were also added. Finally, a further independent *Quantitative* dataset was created by measuring the MIC on a small number of missense mutations to test if our models can predict the magnitude of the effect.

**Table 1.** Description of datasets employed in this study

Dataset	Phenotype	# Isolates	# Non-redundant missense mutations
Train	R/S	n/a	464
Test	R/S	n/a	200
Validation	R/U/S	24 231	367 (199 with an R/S phenotype)
Quantitative	MIC	71	57

R, resistant to antibiotic; S, susceptible; U, inconsistent results.

### Pyrazinamide MIC determination

Isolates used for MIC determination came from the EXIT-RIF study and US CDC. Of the 366 *M. tuberculosis* clinical isolates, 333 were collected as part of a prospective cohort study (‘EXIT-RIF’) between November 2012 and December 2013 in three South African provinces (Free State, Eastern Cape and Gauteng). An *M. tuberculosis* databank housed at the SAMRC Centre for Tuberculosis Research, consisting of ∼45 000 drug-resistant isolates collected in the Western Cape province since 2001, was queried to identify isolates containing both pyrazinamide MIC data and *pncA* genotypic data; this produced the remaining 33 *M. tuberculosis* clinical isolates. Isolates that harboured single amino acid substitutions in PncA (39 out of 366 total) were selected for comparison to model predictions. An additional 32 clinical isolates (collected from 2000 to 2008) harbouring single missense mutations in *pncA* came from the culture collection at the Laboratory Branch, Division of Tuberculosis Elimination, US CDC.

All MICs were determined using the non-radiometric BACTEC MGIT 960 method (BD Diagnostic Systems, NJ, USA) with manufacturer-supplied pyrazinamide medium/supplement as previously described.^51^ This system makes use of modified test medium, which supports the growth of mycobacteria at a pH of 5.9. Isolates from the EXIT-RIF study were tested at 100, 75, 50 and 25 mg/L whilst the CDC used pyrazinamide concentrations of 50, 100, 200, 300, 400, 600 and 800 mg/L. A fully susceptible *M. tuberculosis* laboratory strain H37Rv (ATCC 27294) was included as a control for all isolates tested. The resulting 71 isolates contained one of 59 missense mutations; of the 10 mutations measured more than once, 2 had inconsistent phenotypes and were removed, leaving 57 missense mutations, of which 50 were resistant and 7 susceptible.

### Adding pyrazinamide to the experimental structure

Since the *M. tuberculosis* structure of PncA^52^ (PDB: 3PL1) does not contain electron density for pyrazinamide, we first fitted it onto the *Acinetobacter baumannii* PncA structure^53^ and retained the coordinates of pyrazinamide. This was done using the MultiSeq analysis tool in VMD-1.9.4a57, which in turn used STAMP to perform the structural alignment. The root mean square deviation (RMSD) between the C_α_ atoms in 162 residues overlapped in the sequence alignment was 1.22 Å and the RMSD between the C_α_ atoms in the active site (Figure 1c) is 0.75 Å. Taken together these indicate that the structural core of both proteins is similar. The position and orientation of the pyrazinamide molecule is only required for one structural feature, the distance from the pyrazinamide molecule.

### Determination of structural-, chemical- and evolutionary features

A wide range of structural, chemical, thermodynamic and evolutionary features were added.^54^ Structural features included the distances from the Fe^2+^ ion and pyrazinamide molecule, solvent accessibility, backbone angles, secondary structure, temperature factor and depth from the protein surface. The effect on the chemistry was captured by the change in mass, volume, isoelectric point, hydrophobicity, chemistry^55^ and degree of hydrogen bonding. To assess a *pncA* mutation’s impact on the stability of PncA, we added scores from three meta-predictors (RaSP,^56^ mCSM^57^ and DeepDDG^58^). Finally we added MAPP scores, which aims to quantify the evolutionary constraints imposed on a given position in a protein,^59^ and SNAP2 scores. SNAP2 is a neural network trained to predict whether protein mutations are neutral or have a deleterious effect on function.^60^

### Training and reproducibility

Logistic regression (LR), a multi-layer perceptron classifier (NN) and a gradient-boosted decision tree (XB) were all trained as described in our online code and data repository—this also contains saved states of the final models and Python3 Jupyter Notebooks, allowing one to reproduce in a web browser all results and figures.^61^

## Results

### Observed genetic variation in pncA

Since it includes the results of an *in vitro* mutagenesis study, the *Train/Test* dataset captures the most genetic variation in *pncA*. Mutations are observed at every codon bar one (Figure 1a) and all possible amino acids arising from a single nucleotide substitution are observed at several codons. Interestingly, there were a significant number of *pncA* codons where mutations associated with either resistance or susceptibility were seen, confirming that the change in local chemistry introduced by the mutant amino acid is an important factor in determining resistance (Figure 1a). The codons with the greatest mutational diversity in the dataset were all residues involved in active site formation or metal binding, suggesting that, consistent with our hypothesis, loss or alteration of these functions is a common mechanism for gaining pyrazinamide resistance. Indeed, previous studies have noted a negative correlation between a mutation’s distance from the active site and its tendency to cause resistance.^30,38,52^

### Clinically observed association between genetic variation in pncA and pyrazinamide resistance

Overall, 3351 samples (14.7%) in the CRyPTIC dataset are resistant to pyrazinamide and 6851 samples have one or more genetic variants in either the promoter and/or ORF of *pncA*. The majority (6622 samples; 96.7%) have a single genetic variant with 93.9% (6221 samples) of these being substitutions. The remaining 401 samples (6.1%) contained insertions, deletions and frameshifts and these were strongly associated with resistance (343 samples; 85.5%),^21,39^ consistent with their likely disruption of the PncA enzyme. Most synonymous substitutions [present in 3288 (49.7%) of the single variant strains, Figure 1b] were not associated with resistance; however, seven variants were observed in resistant isolates. S65S (19 resistant isolates) is a phylogenetic SNP present in Lineage 1; however, it is susceptible in 3204 strains, suggesting that these 19 isolates are either phenotyping errors or that there is an alternative mechanism of pyrazinamide resistance at play in these strains. The remaining mutations—R2R, L19L, A46A, D63D, 131V and V155V—are each present a single time (twice for A46A), limiting our ability to associate these variants with resistance. Thus, non-synonymous substitution variants [present in 2766 (41.8%) of single variant strains] appear to be associated with most of the pyrazinamide resistance in *M. tuberculosis*.

### Feature determination using Test/Train dataset

**Figure 2.**
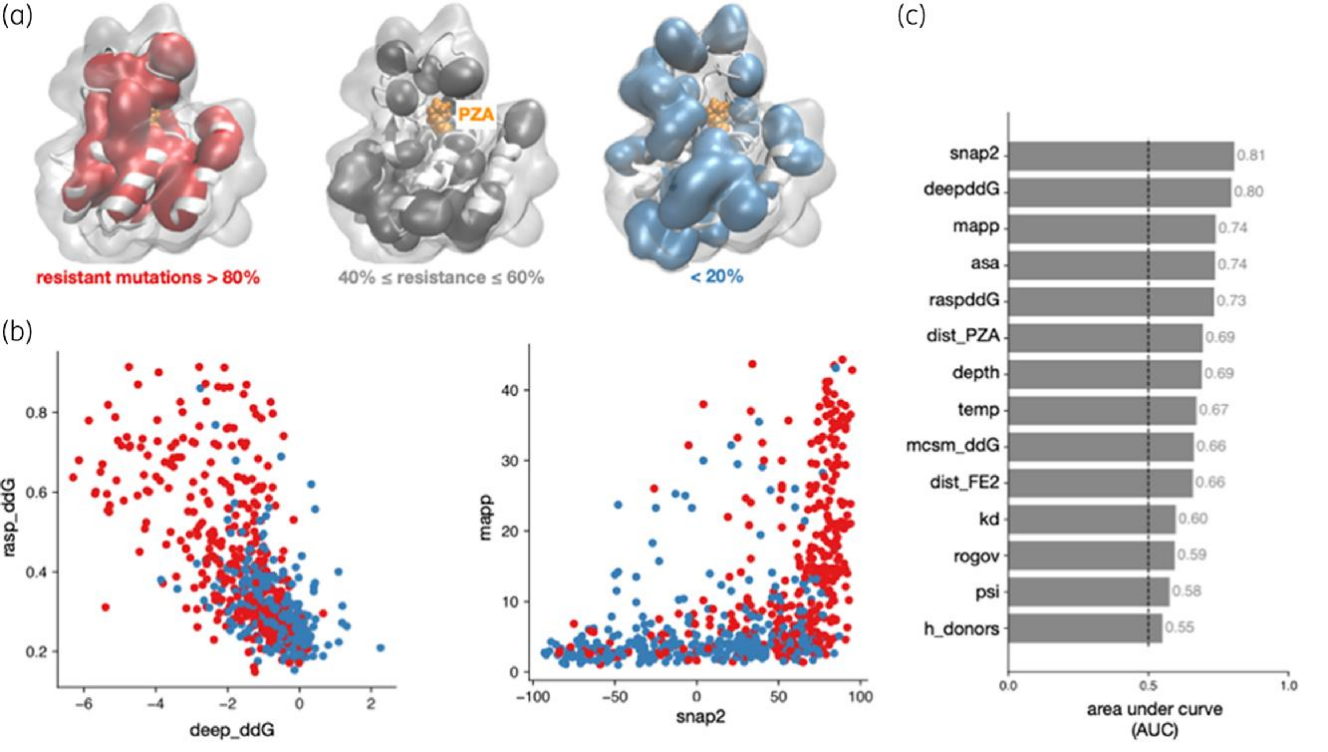
Structural and evolutionary traits correlate with mutational impact on pyrazinamide susceptibility. (a) Amino acids where >80% of mutations confer resistance are more likely to be found in the core of PncA. (b) There is only a moderate correlation between RaSP and DeepDDG, which both predict the effect of a mutation on protein stability, and MAPP and SNAP2. Resistant and susceptible mutations are plotted in red and blue, respectively. (c) The performance of individual features, as measured by the AUC of a univariable logistic regression. The dashed line denotes random guessing.

To understand the structural features that determine a mutation’s effect on pyrazinamide susceptibility, we mapped our combined *Train/Test* dataset onto the PncA structure. No obvious clustering was revealed, consistent with the previously observed distribution of resistant mutations across the gene sequence and protein structure (Figure 1a and c).^13,24,29,30,38^ Examining the PncA structure also suggested that resistant mutations were more likely to be buried in the hydrophobic core of the protein and therefore likely destabilizing, consistent with findings from previous *in vitro* and *in vivo* screens (Figure 2a).^30,38^ Indeed, some pyrazinamide-resistant mutations result in reduced production of functional PncA, perhaps due to impaired protein folding/stability.^38,62^ Despite having a similar learning objective there was only a moderate level of correlation between the different models that predicted the effect of a mutation on the protein stability (Figure 2b). Other more accurate methods exist, but these require several orders of magnitude of computational resource.^63^ Since SNAP2 uses evolutionary information derived from a multiple sequence alignment, one might expect some similarity to MAPP, but again there is only a moderate degree of correlation between the two scores (Figure 2b).

### Machine-learning models accurately predict pyrazinamide resistance

Univariable LR over the derivation dataset revealed that most of the individual predictors were associated with resistance [Figure 2c, Figure S1 (available as Supplementary data at *JAC-AMR* Online)]. The SNAP2 score and DeepDDG protein stability scores proved to be the most discriminatory individual features and six features (change in molecular weight, volume and isoelectric point, along with the secondary structure, ϕ backbone angle and number of hydrogen bond acceptors) were discarded at this point since their AUC lay below an arbitrary threshold of 0.55.

**Figure 3.**
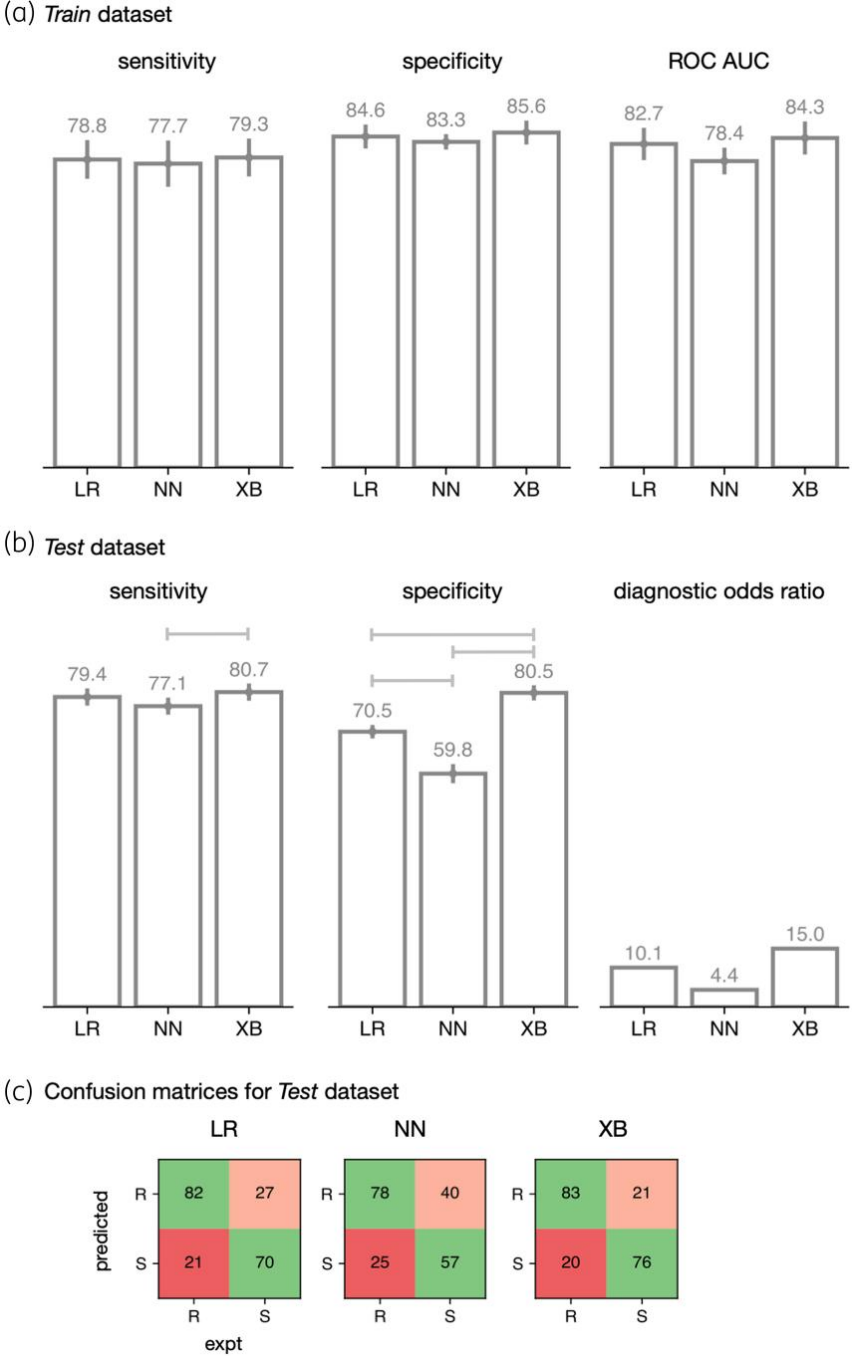
Machine-learning models predict pyrazinamide resistance from structural, chemical and evolutionary features. Performance of logistic regression (LR), a simple neural network (NN) and gradient-boosted decision tree (XB) models on the (a) *Training* and (b) *Test* sets. Error bars represent 95% CIs from bootstrapping (*n* = 10) and brackets indicate a significant difference (*z*-test, *P* < 0.05) (c) Confusion matrices are shown for the *Test* set. VMEs are considered worse than MEs and hence VMEs and MEs are shaded red and pink, respectively.

Following hyperparameter tuning, three different machine-learning models (LR; a gradient-boosted decision tree, XB; and a single layer neural network, NN) were trained on the *Train* dataset using 10-fold cross-validation. All three models performed similarly when applied to the *Train* dataset (Figure 3), with sensitivities of 78%–79% and specificities in the range 83%–86%. As expected, the models performed less well on the *Test* dataset and the XB model had a superior sensitivity (80.7%) to the NN model (77.1%) whilst was indistinguishable from the LR (79.4%) model. The XB model also had a improved specificity (80.5%) relative to the LR or NN models (70.5% and 59.8%, respectively). We conclude that the gradient-boosted decision tree (XB) model performed best since it resulted in the fewest number of resistant samples incorrectly classified as susceptible (so-called very major errors, VMEs) and had the highest diagnostic OR (Figure 3b and c).

### Most residues that were incorrectly predicted as susceptible are surface exposed

**Figure 4.**
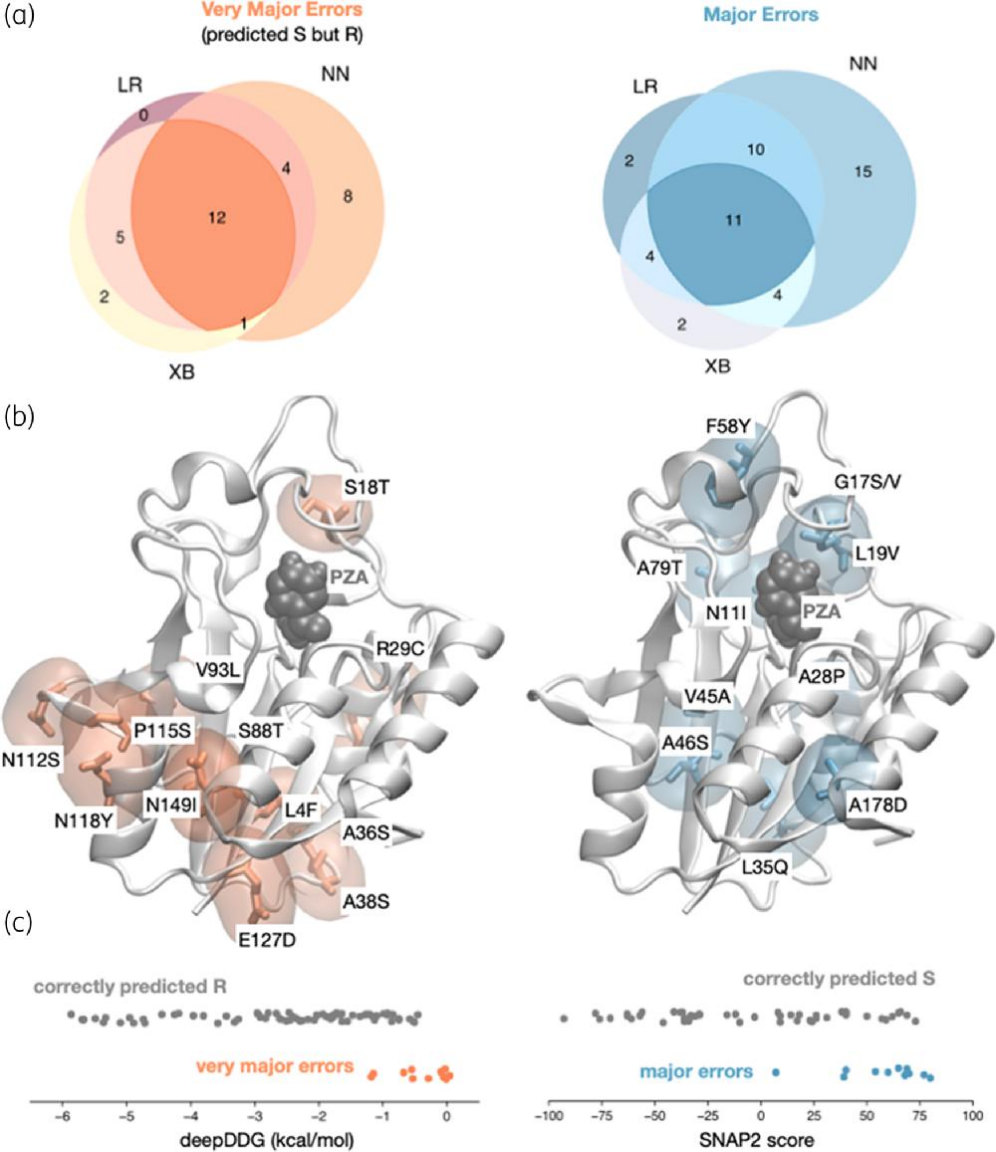
VMEs are concentrated on the surface of PncA. (a) The majority of VMEs and MEs are shared between the three models. (b) PncA with the corresponding residues highlighted where the shared VMEs (orange) and MEs (blue) are found. (c) The shared VMEs and MEs are predicted to have less and more effect, respectively, on the stability of the protein, as exemplified by DeepDDG and the function of the protein, according to SNAP2.

The models predicted 20–25 VMEs and misclassified a further 21–40 susceptible samples as resistant (major errors, MEs). Collectively 12 VMEs and 11 MEs were shared between all three models (Figure 4a). Although the mutations responsible for the shared VMEs were dispersed throughout the protein structure, most (11/12) were surface exposed (Figure 4b). All these mutations were predicted by DeepDDG, mCSM and RaSP to minimally decrease the stability of PncA compared with mutations correctly predicted to confer resistance, suggesting these errors may be partly due to inaccuracies in the predicted free energy change of unfolding (Figure 4c), although other features also contributed (Figure S2). Major errors were also dispersed throughout the protein and were more likely to be buried and to be predicted by SNAP2 to not have a functional effect compared with mutations correctly predicted to have no effect (Figure 4c), although again other features played a part (Figure S2).

Examining the feature importances of the gradient-boosted decision tree (XB) models (Figure S3) shows that whilst all 16 features are incorporated to some extent, the first 4 are all scores from other machine-learning models (MAPP, DeepDDG, RaSP and SNAP2), with the next 4 all being derived from the protein structure (ψ backbone angle, residue depth and residue solvent accessible surface area) or describing the change in chemistry.^55^

### Gradient-boosted decision tree model predictions generalize to a large clinical dataset

A *Validation* dataset was derived from 24 231 *pncA* gene sequences with MGIT antibiotic susceptibility results (Table 1). Most samples contained no mutations in *pncA*: only 4027 samples had 1 of 367 missense mutations. We assume this dataset is representative of the genetic diversity in PncA existing in clinical infections but it is likely biased due to oversampling of outbreak strains and other factors. Until very large unselected clinical datasets are collected and made publicly available, however, it is the best dataset available.

**Figure 5.**
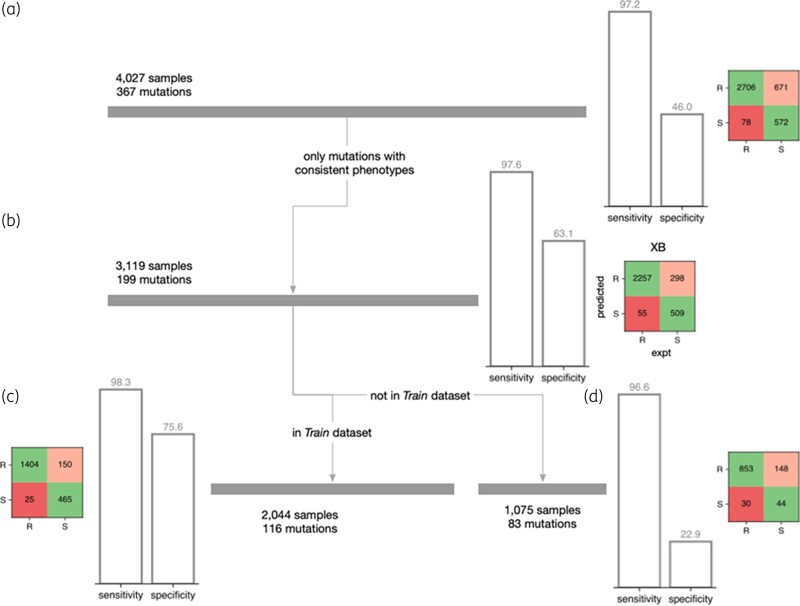
Performance on a real set of clinical samples. (a) Whilst the sensitivity is high, the specificity of the gradient-boosted decision tree model on the *Validation* dataset is lower than observed on the *Test* dataset. (b) Removing samples containing a mutation that has an experimentally inconsistent phenotype increases the specificity. As expected, splitting into samples whose mutation either (c) belongs or (d) does not belong to the *Train* dataset further stratifies performance.

Applying the gradient-boosted decision tree (XB) model to this dataset (Figure 5a) resulted in a high sensitivity (97.2%) but a modest specificity (46.0%). The presence of a substantial number of samples in this dataset (908 samples; 22.5%) contained 1 of 168 (45.8%) mutations that either were only observed once, or whose phenotype varied between isolates was a key contributor to this reduction in performance. Whilst this dataset therefore captures the real-world variability of culture-based phenotypic methods for pyrazinamide susceptibility testing, it is not a good basis on which to assess performance and removing these samples improved the specificity to 63.1% (Figure 5b). Slightly over half (116; 58.3%) of remaining mutations were also present in the *Train* dataset and accounted for 2044 out of the remaining 3119 samples. The predictions for the samples in this group had a sensitivity of 98.3% and a specificity of 75.6%. As expected, the other 83 mutations found in 1075 samples had a lower performance, with the specificity notably being 22.9%. Examining the performance at the level of the mutations (rather than samples) yields a specificity of 48.0%; however, the size of the dataset is now small with only 25 out of 83 mutations having a susceptible phenotype. The XB model also outperforms a previously published model^49^ applied to this same dataset; SUSPECT-PZA achieved a sensitivity of 93.7% and a specificity of 44.3% on the original 4027 samples. Only considering the 199 mutations with a consistent phenotype improved the specificity to 47.7% with a slight fall in sensitivity (92.3%); however, this is less predictive than the performance of the gradient-boosted decision tree.

### Comparison of model predictions with pyrazinamide MICs in vitro

Since it is difficult to assess how much of the discordance in the previous section can be attributed to either error in the measured clinical phenotype or deficiencies in our model, we compared its predictions to MIC data taken from a small but high-quality dataset of 71 *M. tuberculosis* isolates (59 unique missense mutations, quantitative dataset). This also enabled us to test the model’s capacity to predict the *degree* of pyrazinamide resistance conferred by a particular mutation, by comparing the calls and predicted probabilities of our model with the pyrazinamide MICs. Overall, our model correctly predicted the binary (resistant/susceptible) phenotype for 51 of 57 missense mutations in PncA (Figure S4) and, crucially, predicted the correct phenotype for six out of eight mutations that were not in either the *Train* or *Test* datasets. Ultimately, many more samples with a wide range of pyrazinamide MICs will be needed to accurately assess if quantitative prediction is possible for this drug.

## Discussion

We have shown that machine-learning models trained on structural, chemical and evolutionary features can predict whether missense amino acid mutations in *pncA* confer resistance to pyrazinamide, adding to the growing body of work that is exploring different ways of *predicting* antibiotic resistance from genetics.^41–49^ While improvements to the model are necessary to achieve the sensitivity and specificity required for routine clinical use, this work increases our ability to classify rare resistance mutations, thereby potentially increasing the capability of WGS-based diagnostic susceptibility testing to respond to emerging and rare resistance patterns, as well as prioritizing rare resistance mutations for *in vitro* validation. Additionally, improving the classification of susceptible *pncA* mutations will allow us to begin to disentangle the involvement of other genes in pyrazinamide resistance, including determining the effect of mutations in other pyrazinamide resistance-associated genes such as *panD* and *rpsA*.

There are two principal limitations of our approach: (i) since the training set uses a binary resistant/susceptible phenotype, the models can only predict whether a mutation confers high-level resistance (>100 mg/L^64^) or not; and (ii) it can only make predictions for missense mutations in the coding sequence of *pncA*. It is known that genetic variation can lead to small changes in MIC of pyrazinamide and other first-line antitubercular compounds and that, whilst these may not change the binary phenotype, they do affect clinical outcome.^65,66^ In addition, while we have shown that missense mutations represent most of the possible resistant genetic variants in *pncA*, insertions/deletions and non-sense mutations must also be considered, as they are generally associated with resistance. Likewise, promoter mutations that result in reduced transcription of *pncA* will likely also lead to resistance.

Our predictive capabilities will improve with time: the largest potential improvement is likely to come from the availability of larger datasets, preferably with pyrazinamide MICs. Quantitative labels would help delineate mutations that result in an MIC similar to the 100 mg/L breakpoint as one suspects that this effect is the reason why many mutations test inconsistently in the laboratory, which has complicated both our training and validation. New machine-learning approaches and better general-purpose predictors, especially those that aim to predict the effect of a mutation on protein stability, will no doubt come.

Even before that, predictions made by this or similar models could potentially provide clinicians with an initial estimate of pyrazinamide susceptibility after a novel mutation is observed but before traditional phenotypic testing has been completed. Given the latter can take weeks or even months, this could help guide initial therapy and further antibiotic susceptibility testing. In addition, the putative classification of additional *pncA* mutations potentially enables genetic variants conferring pyrazinamide resistance that do not involve the *pncA* gene to be discovered. The identification of pyrazinamide-susceptible mutations is also crucial, as it has been suggested that any non-synonymous mutation in *pncA* that is not catalogued as susceptible confers resistance, an incorrect assumption that would lead to overprediction of pyrazinamide resistance.^67^

The approach used here should be extensible to any pro-drug system where the enzyme is non-essential, such as delamanid, pretomanid or ethionamide, as well as to pro-drug systems in other pathogens. One promising area for future work is in the anti-tubercular bedaquiline, where resistance is caused in part by mutations in a transcriptional repressor (*Rv0678*) that causes loss of DNA binding and up-regulation of efflux pumps.^68,69^ Predictive methods, as shown here, will help accelerate the rate at which WGS approaches move to the forefront of global TB control efforts.

## Supplementary Material

dlae037_Supplementary_Data

## Supplementary data

Figures S1 to S4 are available as Supplementary data at *JAC-AMR* Online.
