# Supplementary material for: Prediction of pyrazinamide resistance in Mycobacterium tuberculosis using structure-based machine-learning approaches
Source: JAC Antimicrob Resist. 2024 Mar 18;6(2):dlae037. doi: 10.1093/jacamr/dlae037 (PMC10946228; doi:10.1093/jacamr/dlae037)
Supplement: dlae037_Supplementary_Data [file dlae037_supplementary_data.docx]

**Prediction of pyrazinamide resistance in *Mycobacterium tuberculosis* using structure-based machine learning approaches.**

Joshua J CARTER^1^, Timothy M WALKER^1^, A Sarah WALKER^1,2^, Michael G. WHITFIELD^3‡^, Glenn P. MORLOCK^4^, Charlotte. I. LYNCH^1^, Dylan ADLARD^1^, Timothy EA PETO^1,2^, James E. POSEY^4^, Derrick W CROOK^1,2,5^, Philip W FOWLER^1,2*^

^1^ Nuffield Department of Medicine, University of Oxford, John Radcliffe Hospital, Headley Way, Oxford, OX3 9DU, UK

^2^ National Institute of Health Research Oxford Biomedical Research Centre, John Radcliffe Hospital, Headley Way, Oxford, OX3 9DU, UK

^3^ Division of Molecular Biology and Human Genetics, SAMRC Centre for Tuberculosis Research, DST/NRF Centre of Excellence for Biomedical Tuberculosis Research, Faculty of Medicine and Health Sciences, Stellenbosch University, Tygerberg, South Africa

^4^ Division of Tuberculosis Elimination, National Center for HIV/AIDS, Viral Hepatitis, STD, and TB Prevention, Centers for Disease Control and Prevention, Atlanta, Georgia, United States

^5^ NIHR Health Protection Research Unit in Healthcare Associated Infection and Antimicrobial Resistance at University of Oxford in partnership with Public Health England, Oxford, UK

‡ on behalf of the “EXIT-RIF” investigators: Prof Robin M Warren, Prof Annelies van Rie, Prof Lesley Scott, Prof Wendy Stevens

Running title: Predicting pyrazinamide resistance by machine learning

^*^ Corresponding author and Lead Contact: [philip.fowler@ndm.ox.ac.uk](mailto:philip.fowler@ndm.ox.ac.uk), @philipwfowler

**Figure S1** Related to Figure 2. Distributions of structural features vary with label. Resistant (red) and susceptible (blue) features are shown with a p-value calculated by a Mann-Whitney U test. The distributions of some features are (A) significantly different between resistance and susceptible mutations whilst others (B) are not significantly different. For clarity not all features are shown. (C) Receiver-operator characteristic curves for all features with an area under the curve > 0.55 after training a logistic regression model on each feature in turn.

**Figure S2.** Related to Figure 4. The prediction of the (**A**) very major errors (orange) and (**B**) major errors (blue) shared between the three machine learning models is driven by a number of features.

**Figure S3.** The gradient-boosted decision tree model makes use of a wide range of features.

**Figure S4***.* The gradient-boosted decision tree model correctly predicts 51 out of 57 mutations, however the dataset contains mainly resistant-conferring mutations. The epidemiological cutoff is the highest minimum inhibitory concentration observed within a phenotypically wild-type population; here that is 100 mg/L and is represented by the dashed line, however this has been shifted to make it clear that the four samples with an MIC of 100 mg/L are experimentally susceptible.
